# Quantifying the development of user-generated art during 2001–2010

**DOI:** 10.1371/journal.pone.0175350

**Published:** 2017-08-08

**Authors:** Mehrdad Yazdani, Jay Chow, Lev Manovich

**Affiliations:** 1 California Institute for Telecommunications and Information Technology’s Qualcomm Institute, University of California San Diego, San Diego, California, United States of America; 2 Katana, San Diego, California, United States of America; 3 Computer Science, The Graduate Center at the City University of New York, New York, United States of America; Banner Alzheimer’s Institute, UNITED STATES

## Abstract

One of the main questions in the humanities is how cultures and artistic expressions change over time. While a number of researchers have used quantitative computational methods to study historical changes in literature, music, and cinema, our paper offers the first quantitative analysis of historical changes in visual art created by users of a social online network. We propose a number of computational methods for the analysis of temporal development of art images. We then apply these methods to a sample of 270,000 artworks created between 2001 and 2010 by users of the largest social network for art—DeviantArt (www.deviantart.com). We investigate changes in subjects, techniques, sizes, proportions and also selected visual characteristics of images. Because these artworks are classified by their creators into two general categories—Traditional Art and Digital Art—we are also able to investigate if the use of digital tools has had a significant effect on the content and form of artworks. Our analysis reveals a number of gradual and systematic changes over a ten-year period in artworks belonging to both categories.

## Introduction

Over the last decade, researchers in computer science, social computing and computational social science have published numerous papers analyzing large social and cultural datasets using computational methods. The rise of social networks and media sharing sites in the middle of 2000’s and the availability of their data was one of the main catalysts for the growth of this research. While this work with social media data addressed many questions important for the social sciences, it did not yet addresses the fundamental concern of the humanities: the study of historical changes over significant time periods in human artifacts, norms, ideas, and other dimensions of human culture. Because social media data is so recent, it was not useful for the study of social and cultural changes. To use the terms from linguistics, computational studies of social networks have focused more on synchronic as opposed to diachronic dimension—i.e., the analysis of the system in a given moment rather than its historical evolution. So while we gained in size and granularity of available cultural content and behavior to study, we lost the historical depth of traditional humanities.

Of course, the applications of computational quantitative methods for cultural analysis is not limited to recently available social networks data. They can be also applied to the gradually growing digitized collections of analog cultural artifacts from the past, such as books, movies and TV programs, songs, or art images. The new field of digital humanities that developed in the late 2000s started to take advantage of the digitization of printed texts. There are already a number of interesting studies of historical patterns using large collections of books and newspapers from the last few centuries. The analysis of historical changes in the use of concepts and names using a sample of a few million of digitized books from Google Books is a well-known example of this research [1]. However, so far no one has yet used computational methods to analyze historical changes in large datasets of visual art images, even though such digitized collections have been available for some time. Similarly, no one has yet taken advantage of the massive numbers of born-digital visual artworks available on social networks since 2004, and blogs and web sites since middle of the 1990’s to analyze changes in their content and style over time. We use the term “visual art” in this paper to refer not only to the works created by artists with art degrees circulating within the professional art world, but also works created by non-professional and semi-professional artists, and also photography, illustration, graphic design, motion graphics, and other types of images and video created by authors with art or design training.

To the best of our knowledge, our paper is the first to quantitatively analyze historical changes across hundreds of thousands of art images. We propose a number of general computational methods suitable for quantifying historical development in many kinds of visual art. We then apply our methods to a large real-world dataset containing 270,000 images created between 2000 and 2010 by users of DeviantArt (www.deviantart.com), the most popular social network for amateur, semi-professional and professional artists during that period.

At the time of this writing, DeviantArt has 35 million members and hundreds of millions of artworks. The description of the network emphasize its democratic character: “We believe that art is for everyone, and we’re creating the cultural context for how it is created, discovered, and shared”. [2].

The diversity of artworks available on this network in terms of subjects, genres, techniques, and tools used is remarkable. This becomes clear after an informal examination of selected images and the intricate system of over 1,700 hierarchically organized categories used by DeviantArt members. We have created two interactive visualization which allows exploration of the complete category tree [3, 4].

This diversity of content and styles makes DeviantArt challenging for qualitative analysis, and justifies the turn to computational methods. If we consider small image collections (hundreds to thousands of images) where visual changes over time are very strong and only limited to a few dimensions, they would be directly visible to the human eye. As examples, we have visualized large numbers of paintings by artists such as Vincent van Gogh, Piet Mondian, and Mark Rothko by sorting digital images of their works by creation dates. These visualizations have shown the kinds and rates of change in the styles of these artists over time. We also created visualizations that show stylistic changes in paintings by Piet Mondrian over many years, and covers of *Time* magazine over eight decades (see [5, 6]). But if art datasets are bigger and more diverse, include works by many authors, and the changes are more subtle and/or affect many dimensions in the same time, direct perceptual examination of selected images or visualizations that sort images by date may no longer help. Therefore, a source such as DeviantArt that contains images with all kinds of content and styles created using many different techniques offers a good motivation for developing and testing computational methods.

We downloaded a random sample of one million art images created between 2001 and 2010 and covering all 14 top level categories of DeviantArt network such as Photography, Design and Interfaces, and Cartoons and Comics. For this study we chose images within two such top level categories: Traditional Art and Digital Art categories. Among one million images in our sample, these two categories contain 270,000 images. These categories and their subcategories (portraits, landscapes, etc.) are most compatible with art genres studied in art history and media theory [7, 8], so we believe that this choice make our study more relevant to the researchers in these humanities fields.

Because we are using a dataset of 270,000 artworks created only within 10 years, we can reliably detect and analyze various temporal patterns. No collection in any art museums has as many images for such a time period. For example, National Gallery in London has only 2,000 paintings covering a period from 13th century until 1900. The biggest digitized collections of professional paintings so far is the *BBC Your Paintings* site [9]. It contains digital images of all 212,000 paintings in all museums in the U.K. created over centuries by artists from many countries. Because the temporal density of such collections is much smaller, systematic study of temporal changes in content and styles is much harder.

The choice of Traditional Art and Digital Art categories of DeviantArt gives us another unique opportunity. We can use these categories to study how the use of digital tools has influenced content and form of art images during 2001–2010 period. During this period, digital tools have gradually become ubiquitous. Therefore, in addition to being the first paper in what we can call call “computational art history”, we also offer the first example of “computational media studies”. Note that the study of different media technologies and their effects on the content and form of visual media is a one of the key concerns of media studies.

In this paper we will discuss the development of DeviantArt categories, the sizes and proportions of images, and their basic visual characteristics. The existing research in art history and media theory does not give us reasons to expect that any of the variables we analyze should change in any particular way during 2001–2010. However, our analysis reveals that many variables have been changing monotonically, i.e. the values of the variables are gradually increasing or decreasing in the same direction over the course of the number of years.

## 1 Related research

Analyzing and explaining differences between historical periods has been central for humanities and social sciences since at least the middle of the 19th century. For example, consider three founders of sociology: Karl Marx, Emile Durkheim, and Max Weber. Karl Marx’s economic theory postulated a number of different modes of production that replaced each other in turn over many centuries [10]. Emile Durkheim described evolution of societies as the move from “mechanical solidarity” to “organic solidarity” [11]. Max Weber proposed that the key differences between modernity and traditional societies were rationalisation, secularisation, and disenchantment [12].

The very names of many humanities fields indicate that they are concerned with history. Besides the discipline of “history” proper, we have art history, architectural history, history of photography, media history, media archeology, music history, history of literature, and so on. As an example, consider art history. Similar to the three founders of sociology, the three founders of academic art history were keenly concerned with historical developments in the arts and crafts. Alois Riegl’s manuscripts of 1890’s analyzed stylistic evolution across the entire history of Western art (*Historical Grammar of the Visual Arts*, published posthumously) [13]. Heinrich Woelfflin’s foundational *Principles of Art History* (1915) contrasted representations of forms in the 16th century and 17th century Italian art [14]. Erwin Panofsky’s *Perspective as Symbolic Form* (1927) examined changes in the understanding and representation of spaces between Antiquity, Middle Ages and Renaissance [15].

In contrast to social sciences, we don’t know a single well-known humanities scholars from the 19th or the 20th century who would use quantitative methods to study historical changes in their fields. But in the early 21st century, this started to change. The 2005 book by literary scholar Franko Moretti [16] and the already mentioned computational analysis of changes in word frequencies across of a few million books digitized by Google [1] motivated other researchers to begin quantitative analysis of historical patterns in various fields of humanities using large datasets. Today, there are publications analyzing changes in literary genres in 18th-19th century [17] and 19th century American newspapers [18]; a study of changes in 500,000 popular songs over last 50 years [19]; analysis of changes in editing and visual characteristics in feature films from 1900s until 2010 [20]; and the analysis of text news archive [21].

There is a sizeable literature analyzing visual art, but it does not addresses the questions of historical change. Many earlier papers focused on classifying artworks. This includes analysis of whether particular artworks of a famous artist were indeed created by this artist, or automatic classification of artworks into classes such as art styles [22]. Similarly, several studies [23–25] focus on developing methods and features for classifying specific artists by identifying unique features for each artists. In [24] a Convolutional Neural Network is used to identify specific artists from a large corpus (over 100,000 artworks from over 6,000 artists). While this Convolutional Neural Network achieves identifying unique characteristics of style for individual artists, how such styles change over time is not addressed. Another related study is [26] that develops measures of creativity in artworks and influence of specific works. More recently, the development of social media and crowd sourcing has enabled researchers to obtain aesthetic judgments of artworks and photographs from large sets of people. This has led to another research direction: modelling and predicting aesthetic judgements using quantified characteristics of images [27]. Many techniques developed in this research on artistic images are relevant for the study of temporal changes in visual art.

## 2 Materials and methods

### 2.1 Metadata analysis

Images do not exist in isolation. Instead, they are surrounded by many kinds of non-visual metadata. This metadata can be divided into a number of standard data types such as numeric, categorical, unstructured text, geo-spatial, and temporal. The examples are names of artists, sizes of artworks, their titles, tags, their descriptions and interpretation in art historical texts, locations of artists places of work, spatial paths of visitors in museums, number of likes or favorites on media sharing sites, and recordings of eye movements, brain activity, or other behavior characteristics of the people experiencing the artworks. In other words, everything which is external to the artworks but is related to their reception, authorship and circulation can be treated as their metadata.

In this paper, we will analyze the following two types of metadata available in our dataset: number of sub-categories within Traditional and Digital Art categories and numbers of images in these sub-categories. Exploring the number of sub-categories may give us insight into the level of cultural interest and variances within the Traditional and Digital Art categories. This would allow us to explore the question if the number of sub-categories can serve as a proxy for cultural variance within each category. In addition to number of sub-categories, we will also analyze width and height of images and their proportions. This would allow us to see if there are any major aesthetic differences between Digital and Traditional Art.

### 2.2 Image features

Along with metadata, we can also extract features from images that describe their content and visual form. The features are numerical summaries of different visual and semantic characteristics of images. In this paper we use so-called “global features” that measure such characteristics across a whole image—for example, mean brightness, saturation and hue, number of edges, properties of textures, and so on.

In machine learning applications, practitioners often use a “black box” solution if it offers the best performance. In our case, we want our methods and their applications to art datasets to be meaningful to art historians, artists, museum professionals, and others who are interested in art. Our goal is to add quantitative methods to other previously developed research methods in the humanities, social sciences and museum and art professions, as opposed to creating an efficient industry application where a black box solution may work best. Although we can imagine such applications—for example, real-time categorization of huge volume of user shared images into types, discovery of their similarity to previously shared images, real-time mapping of world’s digital visual culture, etc. For such applications, efficiency would become quite important. Therefore, in this study we would only use a small number of global interpretable features. These features are statistical summaries (mean and standard deviation) and histograms of hue, saturation and grey scale value of images (abbreviated in the following as HSV).

We calculate these histogram features using the HSV representation of DeviantArt images that are in RGB format [28]. For each image, we have three sets of *n*-bin histograms:

$$\begin{array}{c} \begin{array}{c} H={\left(h_{1},\ldots ,h_{n}\right)}^{T}/\underset{i}{\text{max}}\left(h_{i}\right)\\ S={\left(s_{1},\ldots ,s_{n}\right)}^{T}/\underset{i}{\text{max}}\left(s_{i}\right)\\ V={\left(v_{1},\ldots ,v_{n}\right)}^{T}/\underset{i}{\text{max}}\left(v_{i}\right) \end{array} \end{array}$$ (1)

Each *h*_*i*_, *s*_*i*_, *v*_*i*_ for *i* ∈ (1, …, *n*) indicates a specific bin count in the respective H, S, or V channel. Since images can have different dimensions, the bin counts can vary widely. Therefore, we scale the histogram features by the maximum bin count $\underset{i}{\text{max}}\left(\cdot \right)$ as shown in Eq 1.

Next, we compute the aggregate HSV histogram features by filtering their values with the inverse covariance matrix (that is, a whitening operation [29]) and calculating the mean for each bin for each year. Note that our final results are not sensitive to the choice of scaling and whitening filters. After these steps, we have HSV histogram features for each year in our dataset:

$$\begin{array}{c} \begin{array}{c} H^{t}={\left(\bar{h_{1}^{t}},\ldots ,\bar{h_{n}^{t}}\right)}^{T}\\ S^{t}={\left(\bar{s_{1}^{t}},\ldots ,\bar{s_{n}^{t}}\right)}^{T}\\ V^{t}={\left(\bar{v_{1}^{t}},\ldots ,\bar{v_{n}^{t}}\right)}^{T} \end{array} \end{array}$$ (2)

where *t* ∈ (2001, …, 2010) and $\bar{h_{i}^{t}},\bar{s_{i}^{t}},$ and $\bar{v_{i}^{t}}$ are the mean bin values after whitening the histograms for Hue, Saturation, and Value, respectively.

### 2.3 Pairwise distance correlation between features and targets

We introduce a novel method for analysing temporal changes that we call Pairwise Distance Correlation. In this paper we will use this method for measuring the changes of HSV features of images over time, but the method itself is more general, and can be applied to any set of features and any targets (e.g, external variables). This method is inspired by [30], but we add a new concept: learning the distance metric from data using a Quadratic Program (QP). Other examples of learning distance metrics from data are shown in [31–33].

Consider a set of *N* features and targets {(*f*_1_, *t*_1_), …, (*f*_*N*_, *t*_*N*_)}. The features *f*_*i*_ ∈ ℜ^*n*^ are described above in Eq 2. They are calculated for each year *t*_*i*_ ∈ ℜ ∀*i* ∈ (1, …, *N*). We propose that if there is a relationship between features *f*_*i*_ and targets *t*_*i*_, then this relationship should also be preserved between the distances of pairs of features (*f*_*i*_, *f*_*j*_) and pairs of targets (*t*_*i*_, *t*_*j*_). In our case, if there is a relationship between HSV and years of artworks, then ∃*ϵ*, *δ* such that ‖*f*_*i*_ − *f*_*j*_‖ ≤ *ϵ* and ‖*t*_*i*_ − *t*_*j*_‖ ≤ *ϵ* for |*i* − *j*| ≤ *δ*. Note that this only holds true if the distance between features from images shared further apart in time are larger than the distance between features from images from years that are closer to each other. Effectively we can test this by measuring the correlation between ‖*f*_*i*_ − *f*_*j*_‖ and ‖*t*_*i*_ − *t*_*j*_‖.

To measure this correlation we need to choose an appropriate distance metric. To establish a general metric, we use the weighted 2-norm ${∥f_{i}-f_{j}∥}_{W}^{2}={\left(f_{i}-f_{j}\right)}^{T}W\left(f_{i}-f_{j}\right)$ where the weighting matrix *W* is learned from data. To ensure that we are using a proper metric, the weighting matrix $W\in S_{++}^{n\times n}$ must be positive semi-definite. Furthermore, to learn the weighting matrix using a QP, we impose an additional constraint on the weighting matrix to be diagonal, that is *W* = diag(*w*_1_, …, *w*_*n*_) and *w*_*k*_ ≥ 0 for *k* = 1, …, *k*. To learn the optimal weighting matrix from our data set, we solve the following optimization problem:

$$\begin{array}{ll} \underset{W}{\text{minimize}} & \sum_{i,j}{\left({\left\|f_{i}-f_{j}\right\|}_{W}^{2}-{\left(t_{i}-t_{j}\right)}^{2}\right)}^{2}\\ \text{subject to} & W=\text{diag}\left(w_{1},\ldots ,w_{n}\right)\\ & w_{k}\geq 0,k=1,\ldots ,n. \end{array}$$ (3)

The objective function in Eq 3 is the sum of squares of differences between $∥f_{i}-f_{j}{∥}_{W}^{2}$ and (*t*_*i*_ − *t*_*j*_)^2^ for all (*i*, *j*) feature and target pairs in the data set. Thus, for the data set of *N* examples, there are *N*(*N* − 1)/2 terms in the summation. To solve the optimization problem in Eq 3 we search for the weighting matrix *W* that minimizes this objective function while satisfying the constraints. Note that we can simplify $∥f_{i}-f_{j}{∥}_{W}^{2}$ as

$$\begin{array}{c} \begin{array}{cc} ∥f_{i}-f_{j}{∥}_{W}^{2} & ={\left(f_{i}-f_{j}\right)}^{T}\text{diag}\left(w_{1},\ldots ,w_{n}\right)\left(f_{i}-f_{j}\right)\\ & ={\left(f_{i}-f_{j}\right)}^{T}\text{diag}\left(f_{i}-f_{j}\right)w\\ & =d_{i,j}^{T}w \end{array} \end{array}$$ (4)

where *w* = (*w*_1_, …, *w*_*n*_)^*T*^ and *d*_*i*, *j*_ = diag(*f*_*i*_ − *f*_*j*_)(*f*_*i*_ − *f*_*j*_). We can write Eq 3 equivalently as

$$\begin{array}{ll} \underset{w}{\text{minimize}} & {\sum_{i,j}\left(d_{i,j}^{T}w-{\left(t_{i}-t_{j}\right)}^{2}\right)}^{2}\\ \text{subject to} & w_{k}\geq 0,k=1,\ldots ,n. \end{array}$$ (5)

which is a least squares problem with positive constraints on the unknown weights [34]. This can be solved with numerous QP solvers, for example [35]. The solution to Eq 5 has two benefits: first we are learning the appropriate metric from the data itself to measure distances. Second, the interpretation of the weighting matrix will tell us which dimension of the features are weighted more (and therefore more important) for correlating with targets. Below we will demonstrate applying the weights learned from solving Eq 5 by analyzing HSV features of images from our dataset.

The key difference between Eq 5 and previous metric learning methods [31–33] is that our method is convex, therefore we are guaranteed to find an optimal solution. Another difference, the weights that in Eq 5 is no more than *n* parameters (the inequality constraints also leads to sparse solutions) whereas in general metric learning methods have *O*(*n*^2^) since a full weight matrix is learned. This provides the Eq 5 leading to more interpretable solutions.

### 2.4 Dataset

DeviantArt defines itself as “The world’s largest online art gallery and community”. The network was started in August 2000 and it systematically grew since then. In 2011, it was the 13th largest social network in the world. By March 2013, it had more than 25 million members and 246 million submitted artworks [36] The network is popular with non-professional and semi-professional artists and also art and media students. In contrast, most artists who belong to the commercial art world as represented by big commercial galleries, auction houses, private collectors, art fairs and museums often avoid putting images of their art online, and also would not participate in online community of “amateurs” such as DeviantArt.

The DeviantArt community made available to our lab and our collaborators a random sample of one million artworks from the site for the purpose of academic research. Specifically, they provided us with the list of the artworks’s URLs as well as all the metadata publicly visible for each artwork on the site. We used this list of URLs to download the images. This dataset was also used in a previous study [37]. The one million artworks in the dataset cover the period from the start of the social network in August 2000 until the end of 2010. Because the number of artworks shared in 2000 is small, in this paper we omit this year and use all remaining artworks covering ten years from 2001 to 2010. The artworks in our sample were created by 30,000 artists. A proportion of artists list their gender, age and/or country. This data shows that these artists live in many countries, although the majority are from the U.S. Analysis of gender and age data shows that DeviantArt is not dominated by either gender or particular age range. Since our dataset does not have all artworks from the galleries of these artists, in this study we are not comparing artists to each other, but only analyze the artworks themselves using their metadata and selected visual characteristics. The key characteristic of DeviantArt art collection is its extreme diversity of genres, subject matter, artistic techniques, visual styles and other characteristics. It offers a unique window into contemporary imagination and creativity of aspiring artists from around the world. In contrast, a media sharing service such as Instagram feature mostly photos and until 2015 they all had the same size and proportions. At the same time, only a small proportion of Instagram contributors would identify themselves as serious art photographers. In contrast, DeviantArt contributors consider themselves artists (or artists in training). The extreme diversity of DeviantArt is very valuable, but it also makes it challenging to analyze it computationally. For example, we can’t simply apply standard methods for computational analysis of photographs, since most DeviantArt artworks are not photographs.

Luckily for us, DeviantArt has another important characteristic that significantly helps us to study user-generated artworks shared on this network, despite their semantic and stylistic diversity. This characteristic is the existence of the detailed system of categories used by the members for the identification of their artworks. Most popular media sharing networks use only tags as a way for users to categorize what they share, and since typically a single image or video is assigned multiple tags, this makes it challenging to understand the content of these artifacts from tags alone. While DeviantArt also uses tags, its hierarchical category system is more important. Our exploratory analysis of the dataset of one million artworks has shown that most users understand well the category system and place their artworks within the right subcategories.

The existence of the detailed category system allows us to understand what subjects the artists are interested in, what media and techniques they use, and how this changed over time. While the detection of objects and types of scenes in photos has advanced considerably in the last few years, the automatic identification of subjects of non-photographic art images attracted much less interest and doing this is far from trivial. Given this, the detailed subject subcategories in DeviantArt system give us a unique opportunity to study the semantic universe of contemporary popular art.

Since the start of DeviantArt in 2000, the number of top-level categories and subcategories systematically grew to accommodate the variety of techniques and subjects in artworks submitted by contributors. The category system is organized as a tree, with a number of top-level categories containing further subcategories. By 2011, many “branches” of this tree had up to 6 levels of sub-categories under them, and the total number of all subcategories was over 1,700.

Examining the structure of this category tree, we found that the first and the second level categories divide art by technique: for example, Digital Art/3-Dimensional Art or Traditional Art/Sculpture. (In this and the following examples, we separate the names of the top category and its progressive subcategories by “/”.) Lower level subcategories often describe subject matter of artworks: for example, Digital Art/3-Dimensional Art/Characters/Animals or Traditional Art/Sculpture/Figurative. Overall, we have an encyclopedic picture of contemporary popular artistic universe, reflecting contributions and interests of millions of artists. This detailed category system is one of the most valuable and unique features of the DeviantArt network, because it does not exist in the same organized form anywhere else. (For example, contemporary art museums and art schools typically use less than half a dozen categories to designate their departments.) The analysis of the historical development of the DeviantArt category structure offers us a unique opportunity to understand how popular arts developed during the first decade of the 21st century (see Table 1 for the distribution of the largest subcategories for Digital and Traditional Art in our dataset).

**Table 1:** Largest subcategories (containing more than 2,000 images) within Traditional Art and Digital Art categories in our dataset.

Category name	Number of Images
Digital Art/Fractal Art	34618
Digital Art/Drawings	30630
Digital Art/Paintings and Airbrushing	29394
Digital Art/Photo manipulation	29064
Digital Art/3-Dimensional Art	17374
Digital Art/Miscellaneous	17152
Digital Art/Vector	9087
Digital Art/Pixel Art	4374
Traditional Art/Drawings	45511
Traditional Art/Paintings	30209
Traditional Art/Sculpture	6738
Traditional Art/Mixed Media	6426
Traditional Art/Street Art	4417
Traditional Art/Body Art	2794

However, while the existence of detailed systems of categories describing various artistic techniques, tools, subjects and genres allows us to study the historical development of these important aspects of visual art over a decade, it does not allow us to directly predict the visual characteristics of images. For example, within the “Drawings” subcategory we can find black and white and color works which may have every possible composition and dimensions. Therefore, in addition to studying the development of the category system, we also need to look at the characteristics of the images so we can understand if and how they changed during the ten year period. In this study we use HSV histograms and our described method for their analysis to quantify some of these changes.

## 3 Results and discussion

We have analyzed both selected metadata available for 270,000 art images created by users of DeviantArt between 2001 and 2010, and HSV features extracted from every image. There is no a priori reason to expect that either subjects or visual characteristics of these images change systematically over this period. But if there were any changes, we may expect to find them in Digital Art rather than in Traditional Art. While the tools and techniques used to make art in the latter category stayed constant for many decades, digital tools and platforms (i.e. software such as Photoshop and Painter, and hardware such as desktops, laptops, tablets, and smart phones) were rapidly developing during 2000–2010. Computers were getting faster, screen resolution and the size of RAM were increasing, and storage was getting cheaper, allowing digital artists to create higher resolution images with more complex effects.

The results of our analysis were unexpected. While some aspects of images in Digital Art category did change during the study period, these changes are relatively small. However, we found that characteristics of images in Traditional Art category changed systematically during this period, and the amount of these changes is much larger than for Digital Art. Our analysis also reveals other interesting differences between Digital Art and Traditional Art which were not previously noted by art historians or digital media scholars. In this section, we will go through details of our analysis. We start with changes in the category system, comparing numbers of images and numbers of subcategories within Digital and Traditional Art over time. Next, we will analyze changes in image proportions and their sizes. Finally, we discuss changes in HSV features for Digital Art and Traditional Art images.

### 3.1 Metadata

#### 3.1.1 Categories

**Fig 1:**
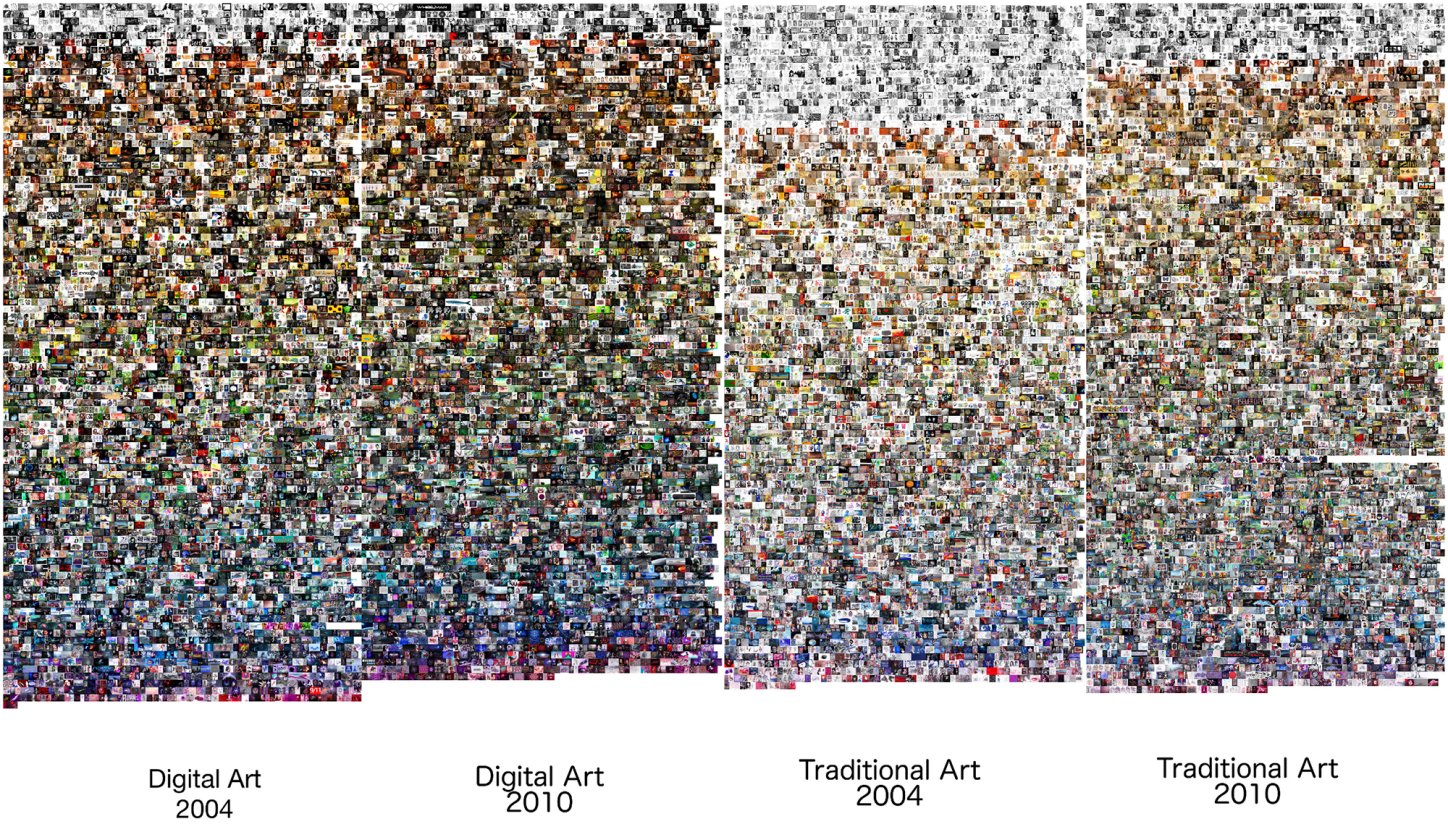
A comparison between 5,000 random samples of artworks shared in 2004 and in 2010 in Digital Art and Traditional Art categories. The images are sorted by hue, with black and white images appearing on top. This explorative visualization suggests that the visual changes in Traditional Art between 2004 and 2010 are larger than the the changes in Digital Art for the same time period. We show in the text how to quantify such changes.

**Fig 2:**
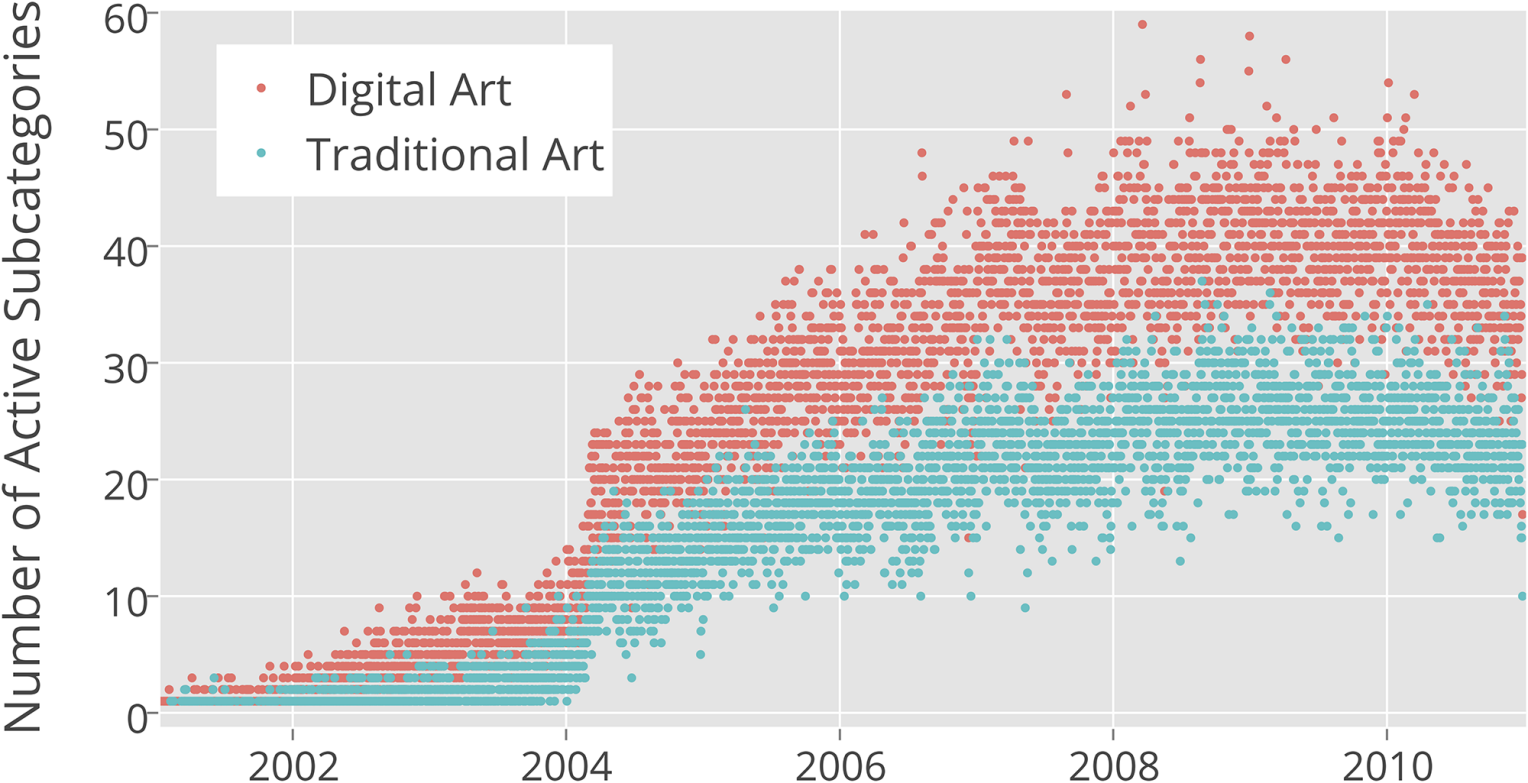
Number of active categories per day.

We compared the numbers of all subcategories for Traditional Art and Digital Art categories during 2001–2010 period. While both categories had a similar growth rate, the number of Digital Art subcategories was always approximately two times larger than the number of Traditional Art subcategories. By the end of 2001, Traditional Art had 10 subcategories, while Digital Art had 22; in 2005, these numbers were 81 and 162; and in 2010, they were 113 and 216. We also examined separately how many of these subcategories were active in any given day (“active” means that users contributed at least one new image in a given subcategory during a 24 hour period). Fig 1 shows a sample of images, and Fig 2 shows the number of active and Traditional Art and Digital Art subcategories per day from 2001 to 2010 in our dataset. While the numbers are smaller than the total number of all subcategories created (not every subcategory was receiving a new image daily), we see the same pattern. The numbers of active subcategories for Digital Art are systematically larger than the numbers of active subcategories for Traditional Art over a ten year time period.

Comparing the total number of images placed by users within these categories once again confirms the same pattern: our sample contains 101,085 images in Traditional Art category, and 177,737 in Digital Art category. (After 2009, the number of newly contributed images became smaller, and correspondingly less categories are active on any given day. We think that the reason for this has to do with rapid growth of other social networks during that time which may be attracting people who previously contributed to DeviantArt.)

#### 3.1.2 Images sizes and proportions

Given the systematic changes in software and hardware for digital art creation during the 2000s, we can expect that Digital Art images on DeviantArt would get progressively larger (as measured horizontally and vertically in pixels). Additional interesting characteristic that we can analyze is the proportion between width and height. While both Traditional Art and Digital Art have portraits and landscapes subcategories, these subcategories contain only a small proportion of total images. Therefore, we did not have any particular expectations about image ratio, not did we had reasons to expect that they will be different for Traditional Art and Digital Art.

**Fig 3:**
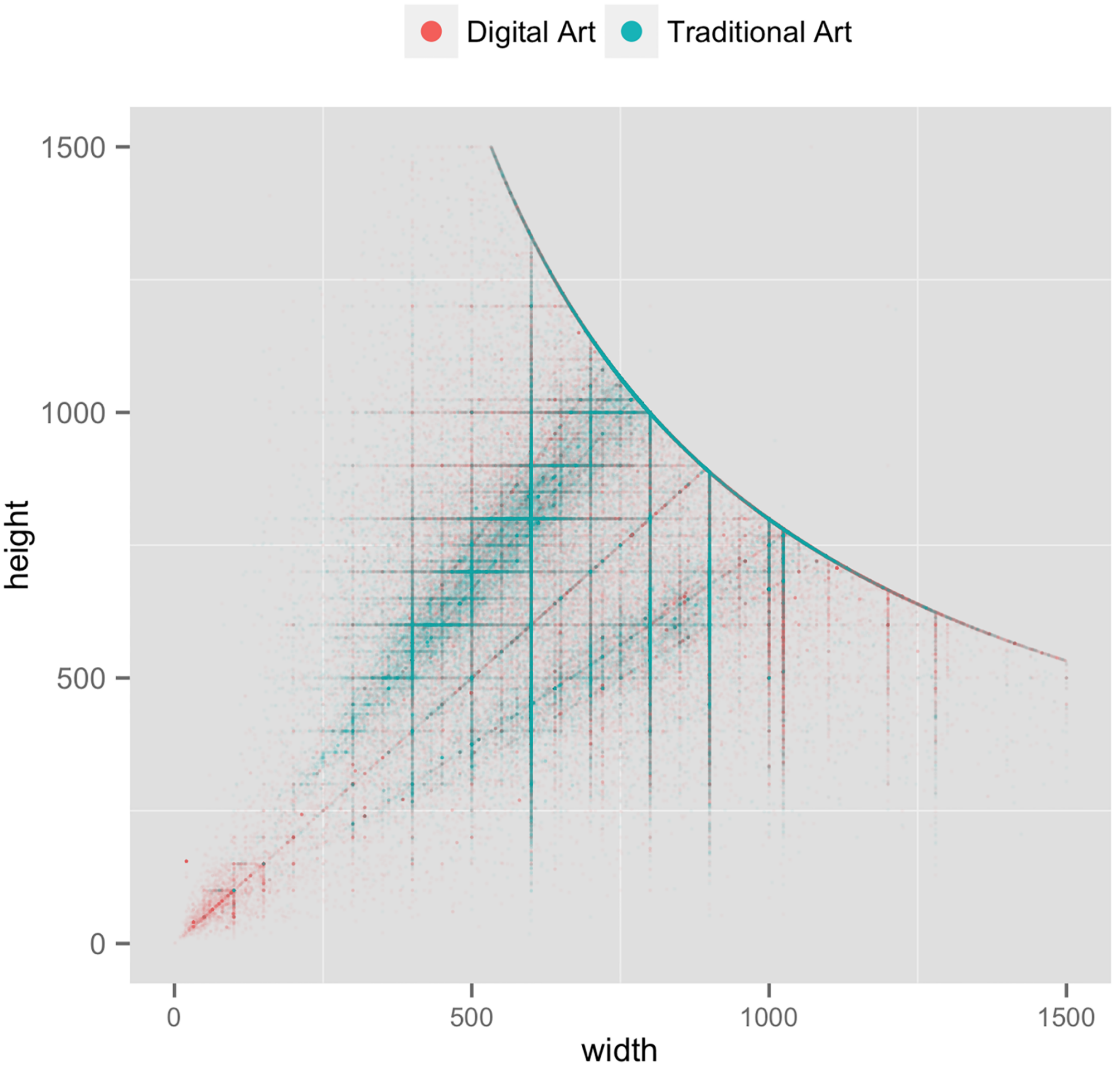
Width and height (in pixels) of the images in Digital Art and Traditional Art categories. Each image is represented as a point.

The analysis of images sizes and proportions led to surprising findings, which can’t be related to any a priori quantitative art historical or media theory analysis. Fig 3 plots width and height (in pixels) of all images in our dataset. Each image is represented by a point. The smooth curve in the plot which acts as the border for combinations of widths and heights is the result of the deliberate limit in DeviantArt web site which prohibits users from adding images bigger than a particular size—so this is not an interesting finding. But the big difference between clustering of points for Traditional and Digital Art is. While the sizes of Digital Art images vary freely, a larger proportion of Traditional Art images have only particular widths and heights combinations. We propose the following explanation for this interesting pattern. When a user of Painter, Photoshop, 3D Studio Max or any other digital authoring software applications creates a new image, she can easily choose any size. Equally important, at any time during the creation process, she can easily crop the image or add a new area. Each operation requires just a few clicks. But the artworks in Traditional Art category typically begin with physical materials bought in an art store, such as stretched canvases and paper. These materials are sold in a fixed number of sizes and proportions, and their size can’t be changed as easily later on.

### 3.2 Image features

In this section we discuss the results of our analysis of changes in HSV features extracted from the images in Digital Art and Traditional Art categories during 2001–2010 period. These features include mean and standard deviation of the Hue, Saturation and Value components of each image. We calculate averages of these two features for all images in each category for every year in our period. In addition, to capture more information, we also extract the 8-bin histograms for each of the HSV components. We then use Principal Component Analysis of these HSV histograms to visualize the yearly changes in Digital Art and Traditional Art. Finally, we use a novel method inspired by the distance correlation statistic and described above to measure the yearly changes in HSV histograms. The advantage of this method is that allows us to analyze temporal changes on many dimensions simultaneously (in the application in this study, we analyze values of all 8 bins of Hue histogram).

#### 3.2.1 HSV mean and standard deviation features

**Fig 4:**
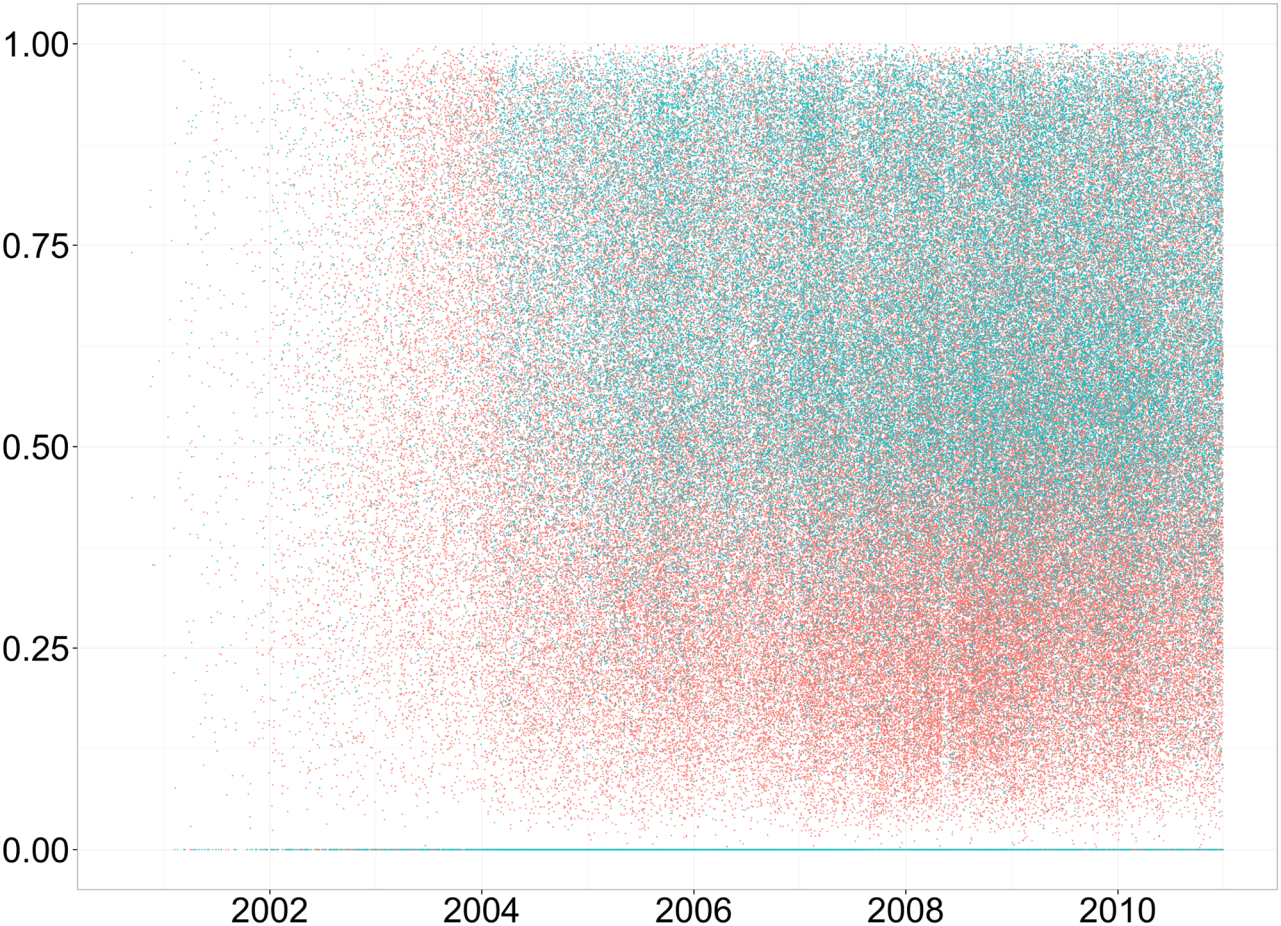
Mean grayscale value of images over time. Red corresponds to Digital Art and green corresponds to Traditional Art as in other figures.

**Fig 5:**
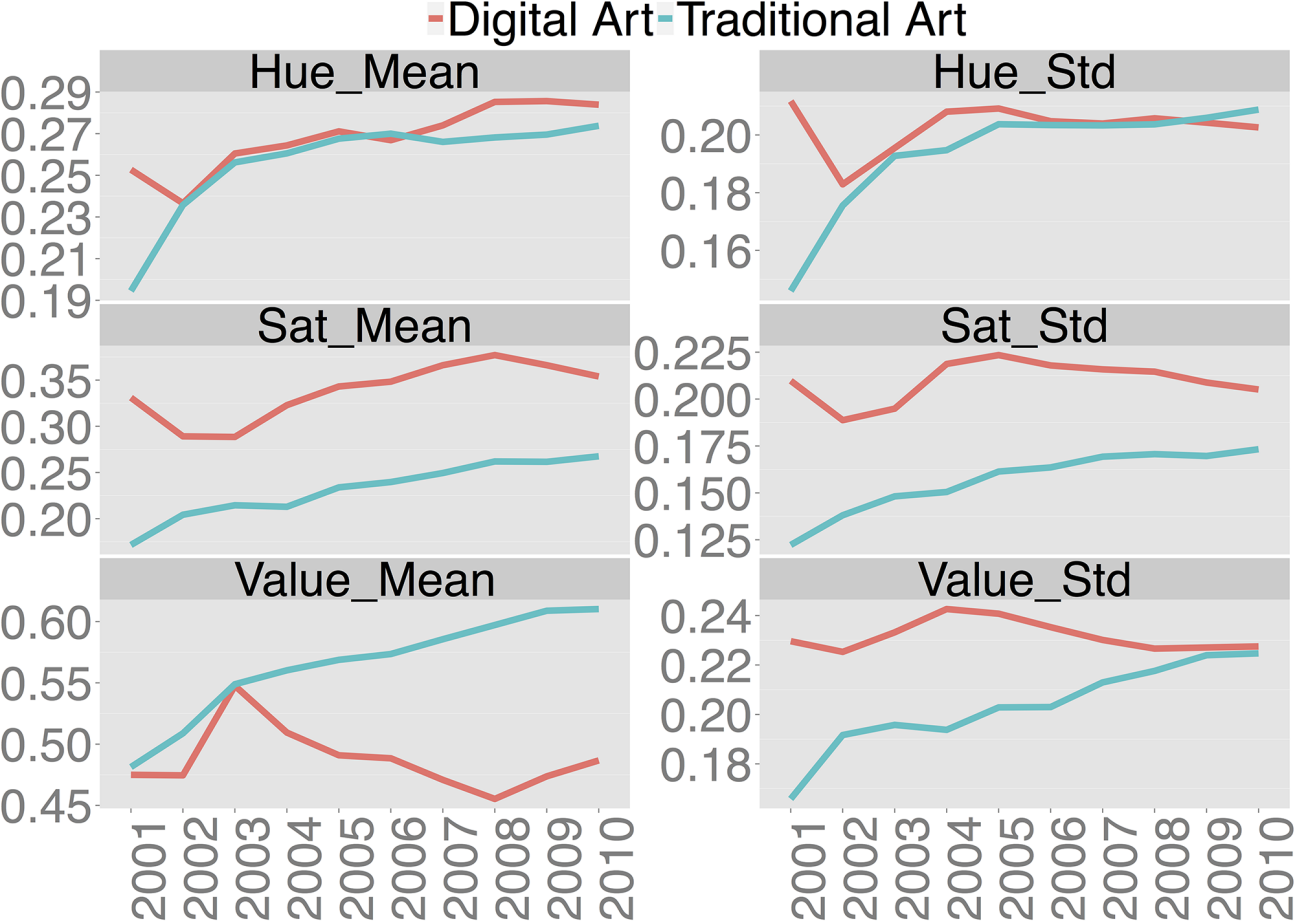
Mean HSV features per year aggregated for all images in Digital Art and in Traditional Art categories.

In Fig 4 we show the scatter plot of the mean Value (i.e., the mean grayscale pixel level) for each artwork in our data set for 2001–2010 period. As this figure shows, Traditional Art has a much more narrow distribution of grayscale values than Digital Art. Fig 5 compares equal size samples (90,000 images) from each category by sorting them on Value mean and Value standard deviation. It also suggest that the variance of Digital Art on greyscale dimension is bigger than that of Traditional Art.

**Fig 6:**
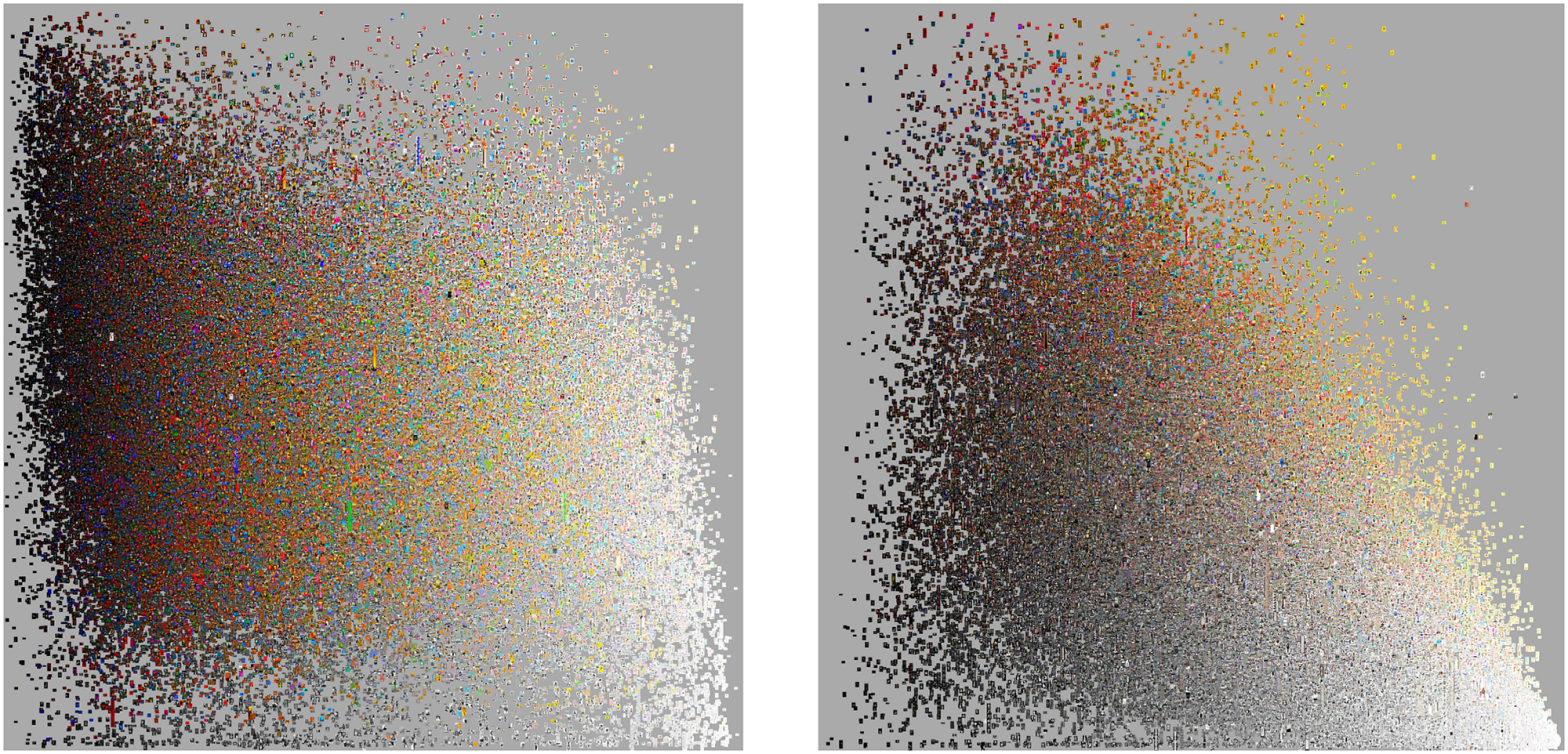
90,000 image sample from Digital Art category (left) and 90,000 image sample from Traditional Art category (right). In each plot, images are sorted by mean Value (x-axis) and mean Saturation (y-axis).

Fig 6 shows the yearly changes in mean and standard deviation of HSV components aggregated for all images in the two categories for every year. As in previous figures, we see that the variance of greyscale values (Value_Std) in Fig 5 for Traditional Art is smaller than for Digital Art. The variances of Saturation levels (Sat_Std) for these two categories are also different, and they follow the same pattern as Value_Std values. One interesting difference is that by 2010 Value_Std for Traditional Art approaches that of Digital Art, but Sat_Std still remains smaller. Our third aggregated feature (Hue_Std) exhibits a different behavior from the other two. For this feature, the values for Digital and Traditional Art remain very similar for most of the years.

These graphs show that each of aggregated HSV image features changes systematically during a ten year period. This is a very interesting finding. Given that we are analyzing 270,000 images created by many different artists using many different materials and software, with different aesthetic goals and styles, there is no reason to expect that the aggregated image characteristics will be changing over time in any systematic way. The presence of the systematic temporal trends in hundreds of thousands of art images is something which would be very hard to see without computer analysis. The patterns such as the ones we report on in this paper show the possibilities of computational art history and justify the need for using computational tools to analyze both contemporary born-digital and historical digitized image collections.

Since the just discussed graphs use simple summary statistics which reduce images to just two numbers (mean and standard deviation), we may suspect that they don’t reveal all details of temporal changes in HSV components. In the next section we discuss our further analysis which uses more informative HSV histogram features.

#### 3.2.2 Principal component analysis of HSV histograms

**Fig 7:**
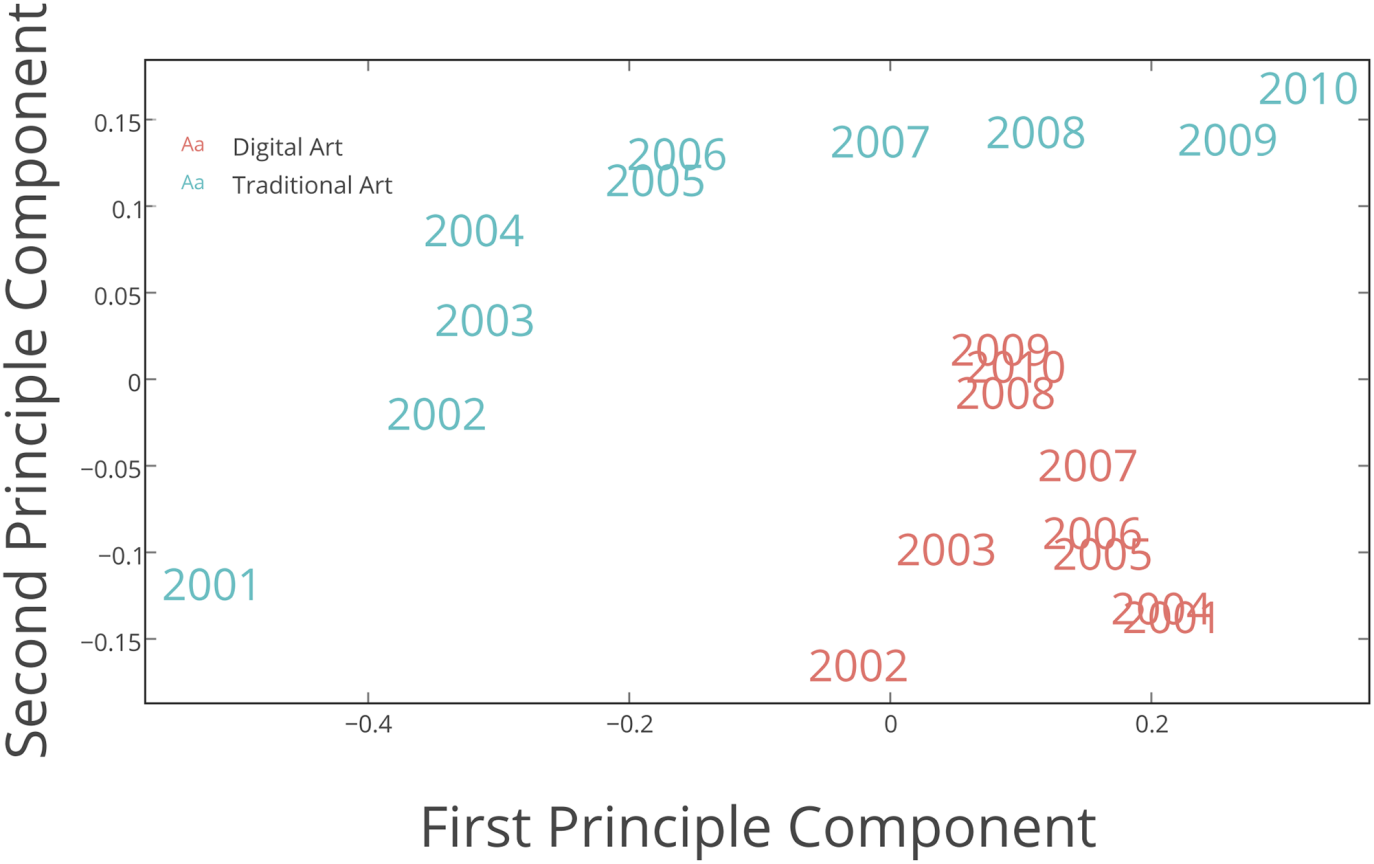
First two PCA components of Hue histogram features.

To analyse changes in images visual characteristics in more detail, we calculate 8-bin histograms for Hue, Saturation and Value of every image. We then compute the average histogram values by aggregating the values of all images for each year and normalize each bin component as shown in Eq 2. To visualize this feature set, we use Principal Component Analysis (PCA). Fig 7 shows the first two Principal Components for the Hue histograms. We use the first two Principal Components since they account for the most variance, and we would like to visualize in 2D the features that we computed in Eq 2. As we can see, Digital Art has more variation along the second component. Traditional Art has significant variation along both first and second components.

We have also tried other metrics and other dimensionality reduction methods (such as Multidimensional Scaling), but our results are remain unaffected. We prefer PCA, however, since the axes in PCA indicate variance in the data and hence are more interpretable than the axes in other methods. Note also that each point in Fig 7 is based on an the aggregated features of all the images in our data for each of the years and subcategories as computed in Eq 2.

This plot suggests that the Hue values of Traditional Art images vary more over time than the Hue values of Digital Art images. Looking more closely, we can see that the changes in Hue values of Traditional Art have two distinct periods. Before 2005, most of changes in Hues of Traditional Art are along the second component. After 2005, most of the changes are along the first component. Digital Art does not have such periods. In fact, while its Hue values change from year to year, they don’t follow an orderly pattern. To quantify these temporal differences and degree of changes, we use Pairwise Distance Correlation method in the next section.

#### 3.2.3 Pairwise distance correlation analysis

**Fig 8:**
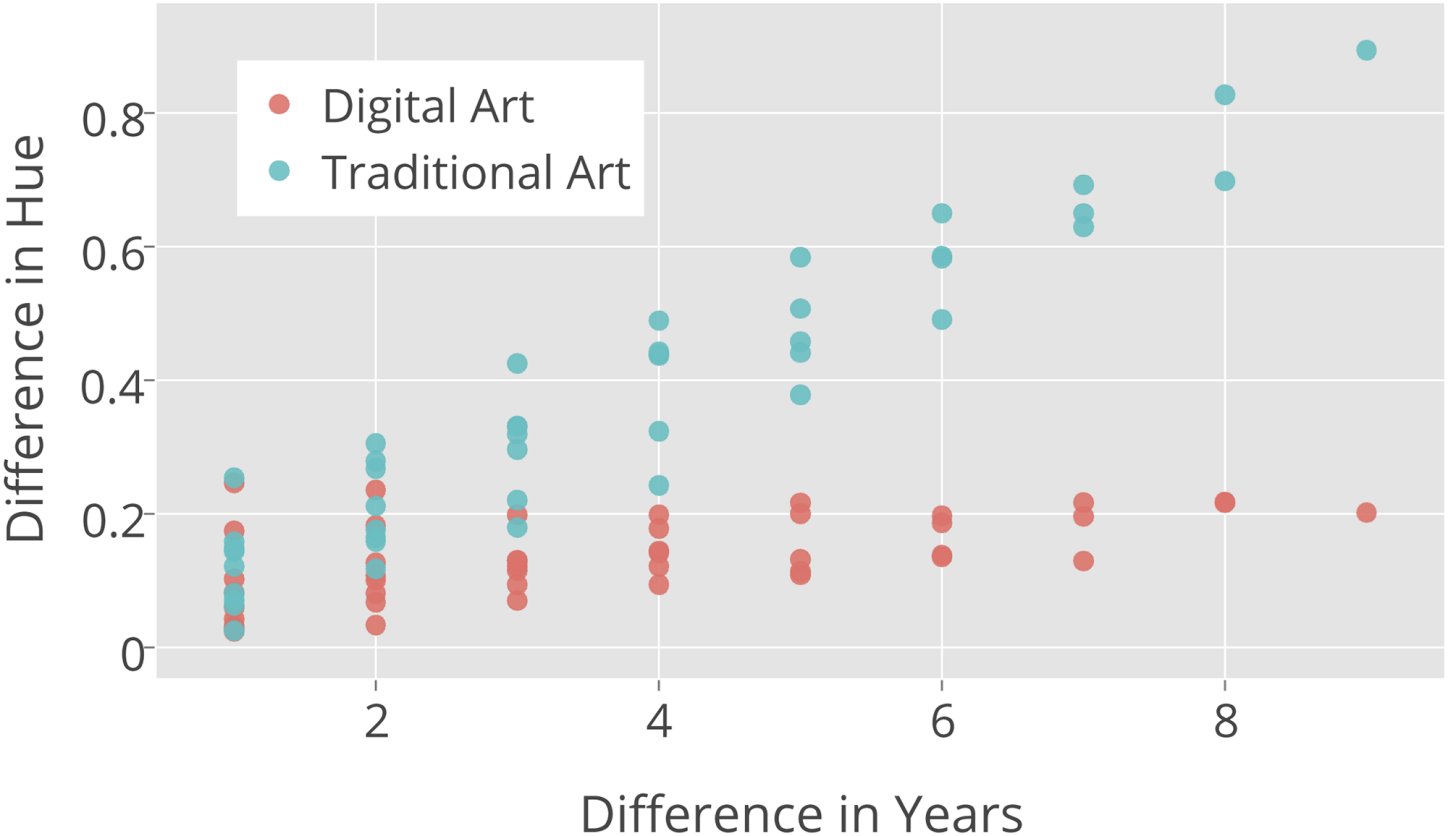
Changes of Hue Feature histogram vs. time differences for Digital Art and Traditional Art images.

Fig 8 shows a scatter plot of the pairwise distances between the average Hue histogram distributions for every year for the Digital Art and Traditional Art categories. Rather than plotting temporal unites such as years on X-axis, we plot paiwise differences between all yearly values. For example, “9” corresponds to only one difference (2001–2010); “8” corresponds to two differences (2001–2009 and 2002–2010); “7” corresponds to four differences, and so on. Y axis show the corresponding aggregated Hue values for each of these temporal differences.

This plot reveals that as the difference in the number of years increases, the distance between the Hue distributions also increases. It also shows that the differences in Hue histograms for Traditional Art over time are significantly greater than for Digital Art.

Fig 1 leads us to the same conclusion but it is uses only a small sample of all 270,000 images and it also does not quantify the amount of changes. While this Figure suggests that the difference are present, the Pairwise Distance Correlation analysis of HSV histogram features allows us to quantify the degree and directions of temporal changes. Table 2 shows the average pairwise distances for H, S, and V features. Note that the pairwise distances for Traditional Art on average are always larger than the corresponding differences for Digital Art.

**Table 2:** Average pairwise distances for HSV features.

Category	Hue	Saturation	Value
Digital Art	0.1350021	0.2179323	0.1955803
Traditional Art	0.3621035	0.2858759	0.2943795

The Hue and Value for Traditional Art is significantly bigger (with *p* ≤ 0.05) than for Digital Art. The Saturation for Traditional Art is bigger than for Digital Art with a *p* = 0.06796. All *p*—values were calculated using a one-sided Kolomogorov-Smirnov test.

Note that the analysis illustrated in Fig 8 used the Euclidean norm to measure distance. As we discussed in the Methods section, it may be more appropriate to use a weighted metric. We introduced a method, formalized in Eq 5, for learning the diagonal weighting matrix from data. More importantly, solving Eq 5 enables us to interpret the results of the Pairwise Distance Correlation method because it tells us which features (in this case these are bins in a histogram) are most important in accounting for temporal changes.

Table 3 shows the weights learned from our data set using only the Hue histograms as computed from Eq 2 for all artworks, and also for Digital Art and Traditional Art categories. The items in bold with asterisks indicate weights that correspond to bins that are most important in accounting for temporal changes in Hue values. In Traditional Art, for example, the bins that have the highest weights are bins 1, 2, and 8. Bin 1 corresponds to black and white images and also images with lots of red. Fig 1 shows that there significantly more Traditional Art black and white images in 2004 than in 2010. Now we can quantify this visual impression by checking the value of the weights for bin 1 for Traditional Art in the table. However, the table also tells us that images with lots of red were also important, although we did not see this in Fig 1.

**Table 3:** Changes in Hue histograms vs time differences calculated using a metric learned from Eq 5.

Bin	Weights: Both Categories	Weights: Digital Art	Weights: Traditional Art
1	3.11E-05	1.20E-04	9.20E+01*
2	1.66E+03*	2.32E+03*	4.78E+02*
3	2.75E+03*	1.46E+09*	2.22E-08
4	6.96E-04	4.86E-04	2.75E-08
5	7.28E-04	8.80E+02*	4.46E-08
6	8.05E-04	1.89E-03	1.17E-08
7	8.19E-04	5.78E+03*	1.05E-08
8	2.14E-04	5.91E-04	2.78E+02*

**Fig 9:**
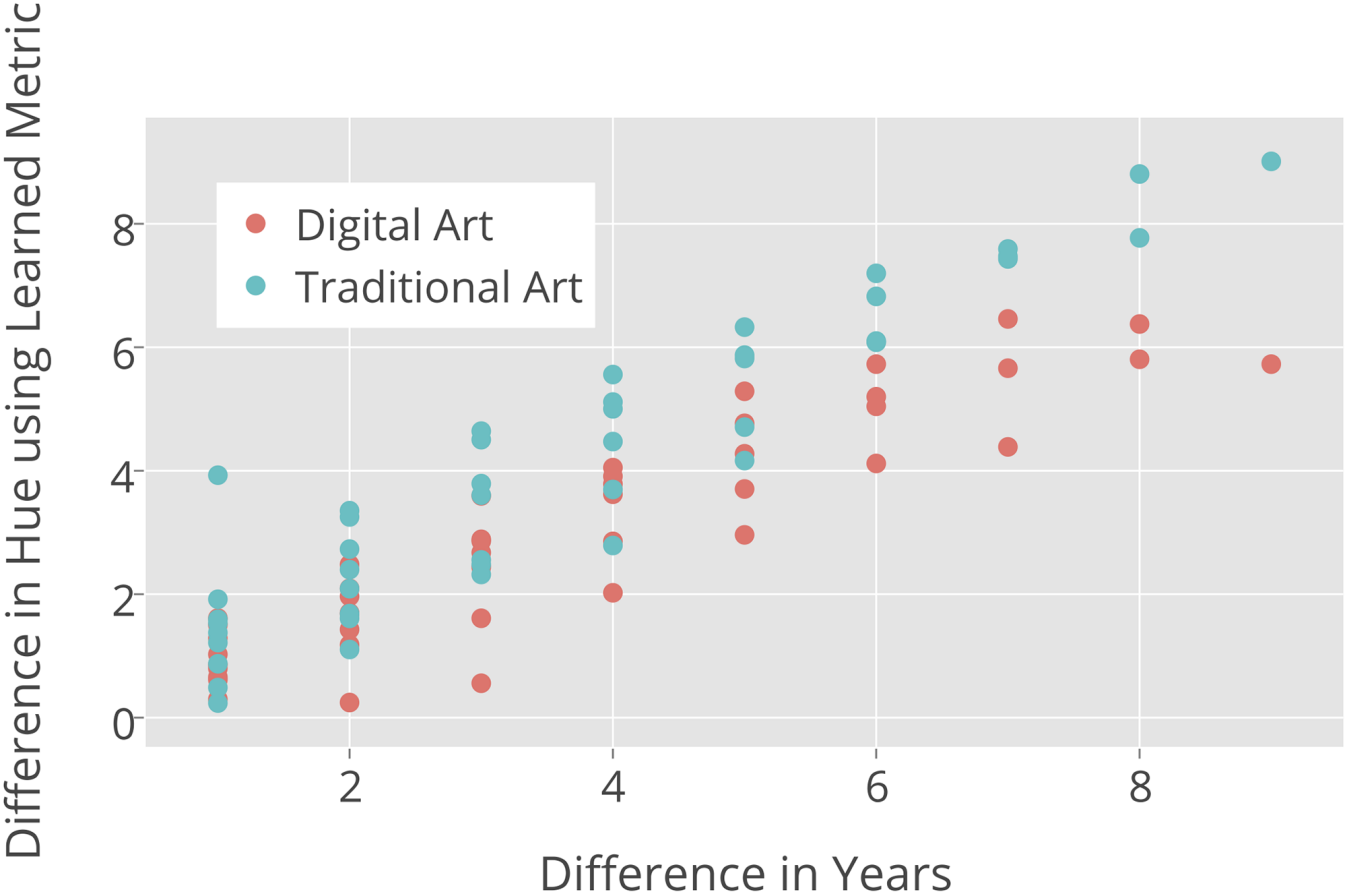
Changes in Hue features over time using a learned metric.

For Digital Art and Traditional Art images combined (left column) bins 2 and 3 have the biggest weights. These bins correspond to yellow, green and yellow hues. Fig 1 also suggests that these hues appear more often in images than other hues for both years chosen for this figure. Fig 9 indicates that if we use the weights calculated using both Digital Art and Traditional Art categories (i.e., effectively using mostly bins 2 and 3), our learned metric shows well how Hues in images change over our time period. However, we can already observe from Fig 1 that perceptually Traditional Art changes more than Digital Art, and our further analysis allowed us to quantify this observation. Therefore, the main advantage of using the the learned metric from Eq 5 is that it allows us to understand which bins are most responsible for temporal changes.

**Fig 10:**
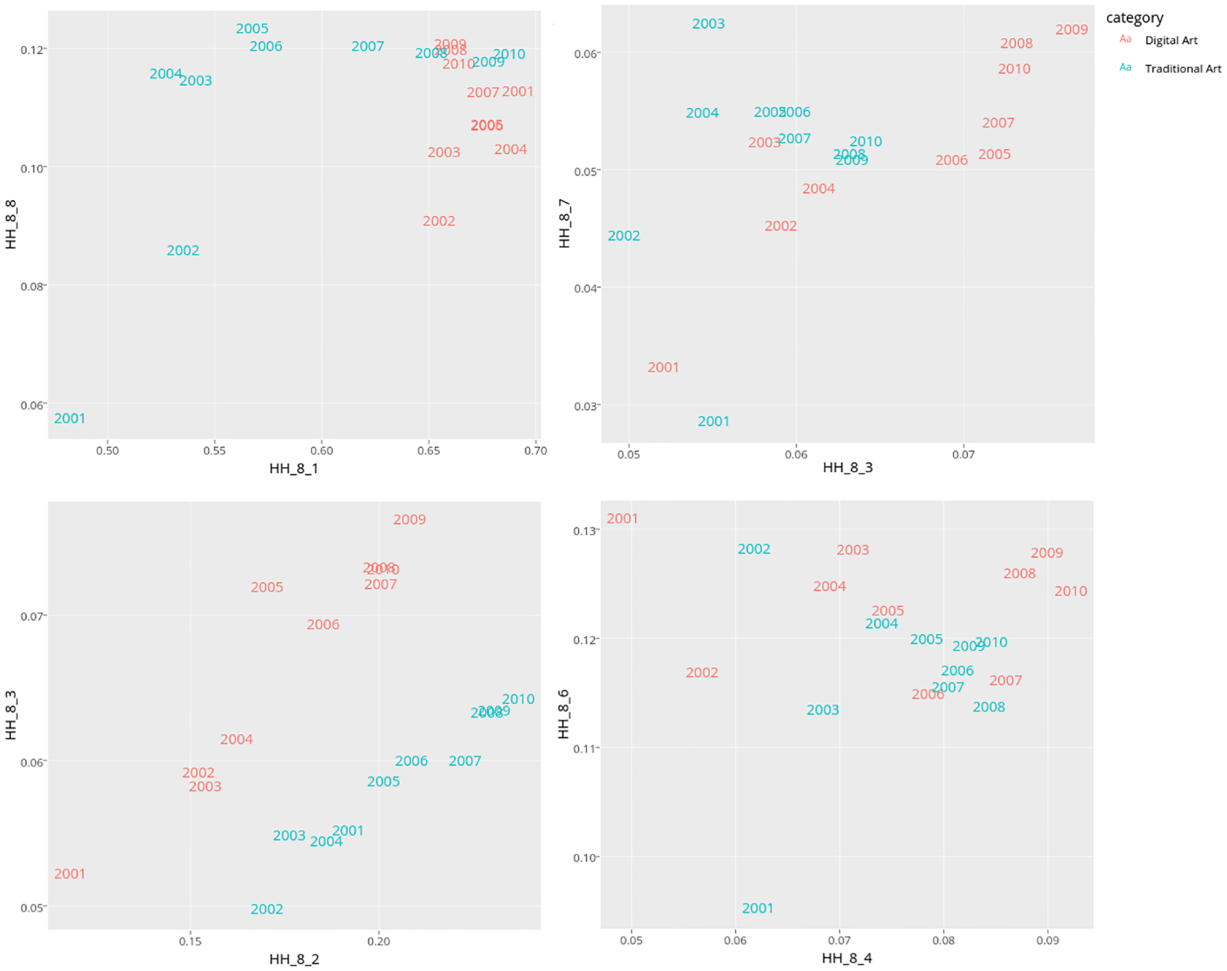
A few samples of bins selected by Eq 5. See text for details.

We next show a sample of the scatter plots of the interesting bins that solving Eq 5 finds. For example, the upper left panel in Fig 10 shows the average hue bins corresponding to bins 1 and 8 corresponding to the important weights for Traditional Art (but not for Digital art) as indicated in Table 3. As shown in this scatter plot, the amount of change that Traditional Art has gone over the 10 year time period of data is significantly larger than Digital Art (this is because Traditional Art covers undergoes more changes than Digital Art does). Furthermore, we also see that Digital Art for bins 1 and 8 shows little relationship to time. This supports our finding that bins 1 and 8 have received small weights for Digital Art as shown in Table 3.

We see a similar result but the counterpart to Traditional Art with bins 3 and 7: both bins have small weights for Traditional Art, but large weights for Digital Art, suggesting that Digital Art has changed more over the 10 year time period than Traditional Art, as verified by the upper right panel in Fig 10. Similarly, the bottom left panel in Fig 10 (corresponding to bins 2 and 3 that have high weight values as shown in Table 3) indicate both Digital Art and Traditional Art have a strong temporal component. On the other hand, the bottom right panel in Fig 10 (corresponding to bins 4 and 6 that have low weight values as shown in Table 3) indicate that neither Digital Art nor Traditional Art have strong temporal components for these particular bins. In summary, our method additionally finds important features to consider and allows us to discard features that are not relevant. As a result, only need to investigate a few interesting features. Otherwise, if we were to exhaustively evaluate every pair, there are 28 combinations of such pairs (and hence grow quadratically with number of features) to consider and therefore not a scalable approach.

#### 3.2.4 Limitations of using color histograms as features

While color histograms are an essential feature used in image processing and computer vision [38–40], as with any feature they have their own unique limitations. One limitation of using color histograms is that the content of the image is destroyed as any ordering of the pixels in an image will create the same histogram. However, color is an important feature for emotional responses to art works and has been shown to be a key perceptual feature for artworks [41]. We therefore think using color histograms captures an important signal that has perceptual significance and interpretability.

Another limitation of using color histograms for Traditional Art, as discussed in [42], is the instrumental variations and noise introduced by the scanners and cameras. While this is a problem for datasets that have been digitized by a small number of institutions, because our data sets were created by thousands of independent users, it is reasonable to assume that the instrument noise is far less systematic and more random than when a small number of institutions digitize large numbers of artworks. Furthermore, if there are still instrument variation issues, we have taken several precautions to minimize such noise contributions. First, instead of using high resolution histograms that account for every bit, our bins are more smoothed out since we are only using 8 bins for the Hue, Saturation, and Value histograms. Second, by averaging the features as we have done so in Eq 2 we have minimized the noise introduced. Finally, we have also computed the standard deviations of each bin for the Hue histograms and have observed that both Digital Art and Traditional Art have comparable standard deviations in every bin for every year. This suggests that the noise contributions from instrument variations is not as large as one expects.

Another concern is that because the color creation process of Digital Art (additive RGB) is fundamentally different from the creation process for Traditional Art (subtractive RYB), this difference may introduce a bias that prevents us from making proper comparisons. However, in this paper we are comparing specific categories (such as Traditional Art or Digital Art) with itself over time. Since our focus is on quantifying the changes of each category over time and not comparing the difference between the two categories, our analysis does not suffer from the differences in color creation processes. Finally, as evidenced by the random sample in Fig 1, we suspect that a bigger source of color changes is the decreasing popularity of sketches in Traditional Art than the color creation process or the instrument noise introduced by cameras and sensors.

## 4 Conclusion and future work

In this paper we propose a number of methods for quantitative analysis of historical changes in visual art. These methods are suitable for large art datasets where direct examination of sample images cannot reveal the presence or the amount of changes. We applied these methods to a sample of 270,000 artworks created between 2001 and 2010 and shared on the DeviantArt network. We looked at the evolution of a category system used by DeviantArt artists to describe genres, techniques and subjects of their artworks, sizes and proportions of artworks, and selected visual characteristics.

The results of our analysis were quite surprising. They could not be predicted using existing qualitative work in art history or media theory. In fact, art historians and media theorists have never directly discussed the aspects of visual art that, as we discovered, were gradually and systematically changing during 2001–2010. Therefore, this study demonstrates that the use of quantitative methods can do more than simply bring precision to the descriptions of previously noted historical changes. It can also reveal changes in the development of art and visual culture we never before considered. These changes may be too subtle and gradual to see unless we use computational analysis of of hundreds of thousands of images, as we did in this paper.

Our sample covers two top-level categories used by DeviantArt artists to classify their artworks: Traditional Art and Digital Art. This allowed us to study if the use of digital tools influenced content and form of artworks created during the 2001–2010 period. The effects of new technologies on art and visual culture is an important topic for contemporary art history and media theory and criticism, but so far nobody analyzed these effects quantitatively using large samples of digital artworks created over time. Furthermore, because of the wild diversity of content of such images, we must control the various factors in our data as muc as possible. For this reason we focus on digital versus traditional art so that we have a control variable that differs by the way the artwork was created.

Our first finding concerns the development of the subcategories of Digital Art and Traditional Art categories. The analysis of growth of numbers of subcategories, the numbers of images shared within them, and the number of active subcategories per day revealed gradual monotonic changes in both Digital Art and Traditional Art subcategories. But while these numbers were gradually increasing for both, the growth rate for the former was twice as big as for the latter. One possible explanation for this difference is that Digital Art has more categories that describe specific digital techniques (Vector Graphics, Pixel Art, 3-Dimensional Art, Fractals, etc.) and artistic “scenes” corresponding to these authoring techniques and specific software tools or applications. (By “scenes” we mean groups of non-professional and semi-professional artists who are passionate about particular digital techniques and authoring applications, and exchange information and learn from each other using publications, local interest groups and online networks such as DeviantArt.) While the tools of Traditional Art did not change for decades, the digital tools kept changing during our analysis period, leading to the formation of such scenes around new tools and new techniques.

This is not dissimilar to how programmers and users of particular software or programming languages participate in user groups, conferences and online forums about these software or languages such as Python, R, and others. In our digital culture, programming tools, languages and software—including tools for art and design—act as new ways of organizing communities. They also become new cultural categories that supplement older categories based on geography or styles. DeviantArt category system reflects this new situation.

Our second finding is that the sizes and proportions of Digital Art images have much more variability than the sizes and proportions of Traditional Art images. This is an interesting manifestation of the key difference in materials of traditional and digital media. The first uses physical surfaces such as paper and canvases that are manufactured and sold in a number of standard sizes. The latter uses malleable pixels and vectors, so an artist can change size and proportions of her artwork in any time.

Our third finding is about changes in basic visual characteristics of artworks—hue, saturation and greyscale value—during 2001–2010 period. There is no a priori reason to assume that these characteristics should change at all. However, aggregating the HSV features for all artworks created in each year, we found systematic monotonic changes in both Traditional and Digital Art categories. The magnitude of the changes was bigger for Traditional Art than Digital Art. We validated these results using three methods: analysis of aggregated global features (mean and standard deviation) over time, Principal Component Analysis using 8-bin feature histograms, and a new Pairwise Distance Correlation method (also using histograms) we introduce in this paper. The last method allowed to understand which histogram bins were responsible for the changes. As discussed earlier, while digital tools change much more rapidly than mostly static traditional tools, our analysis and results suggests that the colors distributions of Traditional Art has been more dynamic than Digital Art. This result is surprising since we would expect, similar to our earlier example programmers and their software packages, we would expect that rapidly changing digital tools would similarly result in rapidly changing Digital Art. Traditionally in art history, technology has been considered a catalyst for major movements and shifts in art. For example, the invention of the paint tube is considered a catalyst of Impressionist art enabling artists to leave studios and work directly from the environment to create a completely new form of art [43]. The reason for this may be that the myriad of options that Digital Tools gives has allowed artist to experiment with completely new forms and compositions (such as the many variations of algorithmic art) and as a result artists creating art are more interested in experimenting with other visual characteristics besides colors.

Finally, we also compared variability of hue, saturation and value characteristics for Traditional Art and Digital Art images. We discovered that Digital Art images have more variability for saturation and value than Traditional Art images (Fig 5). While a recent qualitative media theory book suggested that digital art and design may have higher variability than art and design created with traditional media on some dimensions [7], our study is the first to confirm this using a large image dataset. Moreover, we found particular dimensions of such higher variability (saturation and value) and quantified the differences. As with other temporal changes we discovered, the change in variability on these dimensions for Digital Art turn out to be also monotonic.

The study of art history is one of the major topics in the humanities and a basic question to address in art history is “does art change?” Traditionally art history has been limited to studying specific artists, art works, or sets of artists and art works. Using computational methods, however, allows us to investigate questions of change on a scale that is not feasible by a single scholar. As discussed earlier, in Impressionism it is believed that advances in technology, such as the paint tube, has been crucial for the development of the works of many artists such as Cezanne, Monet, Renoir, etc. However, the number of art works that art historians study in Impressionism is orders of magnitude smaller than the number of works that we have studied for our contemporary era. In this paper we discussed quantifying changes in artworks from a large collection by a large number of artists.

One limitation of our study is that we have not classified the objects of all the artworks in our data sets. Therefore, our analysis is based on an assumption that the object distribution in digital artworks is not different from traditional artwork distributions. We are therefore planning new studies which will both expand our methods for analyzing historical change in visual art and also apply them to other art datasets. For example, in addition to HSV features, we can also look at composition, line orientations, sizes of shapes, and so on. Because of the extreme diversity of techniques, visual styles and subjects of artworks shared on DeviantArt, we did not want to use mid-level or high-level image features in this study since they only would be relevant to narrow subsets of the images. However, if we study historical evolution within a bodies of artworks that use one medium or share one visual language—for example, 20th century modernist black and white photography from NYC’s Museum of Modern Art (MoMA) photography collection, paintings of French Impressionists, or millions of Instagram images shared in cities around the world (to use examples of the datasets we have already prepared for analysis), the use of middle and high-level image features will be appropriate. These can be popular generic features widely used in computer vision (for example, presence of faces, their characteristics, or type of scenes and subjects in photographs), and also more specific “art features” introduced by researchers such as measurements of composition and color palettes.

It is at least as important to build bridges between “traditional” qualitative art and media history and theory fields and quantitative computational data analysis. While our paper is the first to offer quantitative analysis of the historical changes across large sets of art images, a number of researchers already computer techniques to analyze historical changes in other media using large datasets of novels, popular songs, and feature films. Their findings are often original and novel, but often it is not easy to relate them to the types of concepts developed in non-quantitative literary theory, music theory, and cinema studies, respectively. Building bridges between qualitative results and theoretical insights is therefore the key issue if the promise of computational study of cultures and art is to be fully realized. From this point of view, we believe that one of the important aspects of our paper is that it starts making such connections between the qualitative concepts and theories and quantitative analysis for the domain of contemporary visual arts created using both traditional and digital techniques.
